# ALS and CHARGE syndrome: a clinical and genetic study

**DOI:** 10.1007/s13760-018-1029-2

**Published:** 2018-10-13

**Authors:** Carmine Ungaro, Luigi Citrigno, Francesca Trojsi, Teresa Sprovieri, Giulia Gentile, Maria Muglia, Maria Rosaria Monsurrò, Gioacchino Tedeschi, Sebastiano Cavallaro, Francesca Luisa Conforti

**Affiliations:** 1 Institute of Neurological Sciences (ISN), National Research Council, C.da Burga, Mangone, CS Italy; 2 Dipartimento di Scienze Mediche, Chirurgiche, Neurologiche, Metaboliche e dell’Invecchiamento, Università degli Studi della Campania “Luigi Vanvitelli”, Naples, Italy

**Keywords:** ALS, CHARGE, CHD7, NGS

## Abstract

Amyotrophic Lateral Sclerosis and CHARGE syndrome are complex neurological disorders, which never occurred together in the same family and, to date, no putative correlation between them has been described on PubMed Central. Due to our aim was to evaluate the presence of different genetic variants involved in these pathologies, we reported a clinical and genetic description of two sisters affected by these two different disorders. In the CHARGE patient, molecular analysis of the *CHD7* gene revealed the c.8016G >A de novo variant in exon 37. The ALS patient had been screened negative for mutations in *SOD1, TARDBP, FUS*/*TLS, C9orf72 and KIF5A* genes. Anyway, targeted next generation sequencing analysis identified known and unknown genetic variations in 39 ALS-related genes: a total of 380 variants were reported, of which 194 in the ALS patient and 186 in the CHARGE patient. To date, although the results suggest that the occurrence of the two syndromes in the same family is co-incidental rather than based on a causative genetic variant, we could hypothesize that other factors might act as modulators in the pathogenesis of these different phenotypes.

**Electronic supplementary material:**

The online version of this article (10.1007/s13760-018-1029-2) contains supplementary material, which is available to authorized users.

## Introduction

Recent findings in the field of molecular biology have led to significant advances in our understanding of the genetic basis of a number of rare disorders. In particular, most of the neurological diseases have well-established evidence of genetic contributions [1, 2]. Herein, we focused on two complex neurological disorders, amyotrophic lateral sclerosis (ALS) and CHARGE syndrome, whereas until now, CHARGE association and ALS disease never occurred together in the same family and no cases have been reported in the literature. Due to our aim was to evaluate the presence of different genetic variants involved in these pathologies, we reported a clinical and genetic study of a family from South of Italy composed of parents and two daughters affected by ALS and CHARGE syndrome, respectively.

### CHARGE syndrome

The acronym CHARGE was coined in 1981 by Pagon et al. [3], for designing a phenotypically variable, multiorgan genetic disorder, first described in 1979 by Hall et al. [4] involving six cardinal features: ocular *c*olobomas, heart malformation, atresia of the choanae, retardation of growth and/or development, genital abnormalities and ear anomalies with hearing loss [3]. Additional less frequent anomalies including cardiovascular malformation, genital hypoplasia, cleft lip/palate developmental delay, trachea-esophageal fistula, and distinctive facial feature have been described [5]. Most neuroimaging evaluations of CHARGE patients focus on central nervous system findings as important clues to the diagnosis, with more than 90% affected individuals having cranial nerve dysfunction [6]. Consequently, several investigators have suggested that CHARGE syndrome may reflect a polytrophic developmental field defect involving the neural crest cell or the neural tube itself [7]. Anyway, the diagnostic criteria have evolved over time, as described in 2016 by the most recent revision of Hale et al. [8]. Although, immunological problems (similar to DiGeorge syndrome) including severe combined immunodeficiency (SCID) can develop in CHARGE syndrome [9], the frequency and type of immune defects in CHARGE syndrome cases have not been well documented and evaluated, compared with those in DiGeorge syndrome cases [10]. The incidence of CHARGE syndrome (OMIM 21400) was evaluated at 1 in 10,000–15,000 newborns and about 60–70% of children clinically diagnosed with CHARGE have genetic mutations in the *CHD7* gene [11]. *CHD7* gene (MIM 608892) is located on chromosome 8q12.1 starting 61.59 Mb from the p-arm telomere, spanning roughly 188 kb, and consisting of 38 exons, of which the first is non-coding. An 8.994 bp open reading frame and a translation start site in exon 2 have been reported. Over 580 different human pathogenic mutations in *CHD7* have been identified until today (http://www.chd7.org) in all, but one of the 37 coding exons and in some intronic sequences, and predominantly consist of heterozygous single nucleotide variants affecting *CHD7* (Chromodomain Helicase DNA-binding Protein Seven) function. Some mutations are missense but the majority are non-sense and frameshift mutations arising de novo and might result in haploinsufficiency of *CHD7*, thereby producing a truncated protein or causing nonsense mediated RNA decay. There are no mutational hotspots and recurrent mutations are rare. No clear genotype–phenotype correlation has been reported, even among patients with identical *CHD7* mutations although it seems that missense mutations, in general, are associated with a milder phenotype [12]. To date, penetrance in patients with *CHD7* pathogenic variants is of 100%. Due to 97% of *CHD7* mutations are de novo, CHARGE syndrome usually occurs as a new autosomal dominant condition, with variable expressivity and no family history [8]. CHD7 is a 2997 amino acid protein belonging to the chromodomain helicase DNA-binding (CHD) superfamily, which groups ATP-dependent chromatin remodelling enzymes; it is composed of: N-terminal tandem chromodomains (chromatin organization modifier), a central helicase domain, a DNA binding/SANT domain and two C-terminal BRK domains [11]. These proteins share the conserved Snf2 helicase-like ATPase domain catalyzing the translocation of nucleosomes along DNA in chromatin, presumably to modulate access of transcriptional regulators. An altered chromatin structure due to inefficient binding of the CHD7-truncated protein to H3K4me may have a possible (potential) role on epigenetic factors. In fact, recently it was found that CHD7 binds to hypomethylated rDNA and could be acting as a positive regulator of rRNA synthesis [13]. So that, it is required for the maintenance of open chromatin and thus activation of genes essential for granule neuron differentiation, as well as interactions with other cells during embryogenesis.

### Amyotrophic lateral sclerosis

Amyotrophic lateral sclerosis, also known as Lou Gehrig’s disease or Charcot’s disease, is a fatal adult-onset disorder characterized by progressive damage of lower and upper motor neurons that leads to motor paralysis resulting in death due to respiratory failure, with a mean survival of about 3 years following onset of symptoms [14]. Frontotemporal cognitive impairment is present in up to 50% of patients and dementia may occur in 5%. The aetiology of ALS is not well understood, but the disease is considered to be a result of the interplay between genetic and environmental factors [15].

The typical age at onset is between 50 and 60 years, and the global incidence is 1–2 new cases per 100,000 individuals every year, with male sex, increasing age and hereditary disposition being the main risk factors [16]. The majority of cases are sporadic (sALS), whereas about 5–10% of cases show a known genetic basis, having a first- or second-degree relative with the disease suggestive of familial inheritance of ALS (fALS) [17]. The symptoms and pathology of fALS patients resemble those of patients with sporadic form of ALS, suggesting that the mechanisms of neurodegeneration share common pathways. There is no effective cure for ALS, though riluzole slightly prolongs survival and the recently approved edaravone seems to slow down disease progression in a subset of patients when administered early after onset [18].

It is now widely recognized that ALS is a complex disease characterized by a high degree of genetic heterogeneity in which a constellation of causative genes and risk factors have been identified [19, 20]. More than 50% of fALS has been attributed to pathogenic mutations in four major ALS genes, *SOD1, TARDBP, FUS* and *C9orf72*. In European-based populations, more than 180 different mutations in *SOD1* (MIM 105400; NM_000454) have been reported, and account for between 12% and 20% of ALS families *TARDBP* (MIM 612069; NM_007375) and *FUS* (MIM 608030; NM_004960) mutations each account for approximately 4% of fALS [21]. The most common known cause of familial and sporadic ALS and FTD is the expansion of an intronic hexanucleotide repeat in *C9orf72* (MIM 105550; NM_018325) [22], which accounts for approximately 40% of ALS families and 7% of sporadic patients. In addition to these major ALS genes, over 50 additional genes have also been reported as linked, or associated with, familial and sporadic ALS and among these, the very recently discovered novel gene *KIF5A* [23, 24].

## Materials and methods

### Clinical features of the probands

**Fig. 1:**
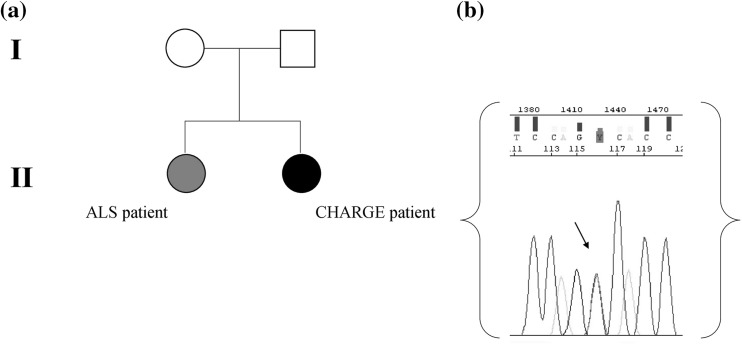
**a** The pedigree for the family; **b** electropherogram showing the mutation detected in *CHD7*. Arrow indicates the site of mutation

The pedigree for the family is presented in Fig. 1. The clinical characteristics of the two sisters, born from non-consanguineous healthy parents, are discussed below. The CHARGE patient, a full-termed 41-year-old female, presented all typical CHARGE syndrome defects, such as bilateral coloboma, choanal atresia, congenital heart disease (patent ductus arteriosus), mild retardation of growth and developmental, mild neurosensory hearing loss and middle ear and ossicular anomalies, micrognathia, facial dysmorphism, renal ptosis, dorsal scoliosis, and genital hypoplasia. No brain malformation was discovered and cognitive abilities were normal. Clinical diagnosis of the ALS patient, a full-termed 43-year-old female, was performed according to the El Escorial revised criteria [25]. ALS was classified as sporadic and no other family members was reported to be clinically affected in the same pedigree. The neurological examination showed bulbar onset at the age of 36, weakness and muscular atrophy in upper limbs, respiratory insufficiency and dysphagia. Bulbar symptoms progressed with weakness and atrophy. No signs of cognitive impairment were revealed.

### Genetic testing

Blood specimens were collected from the family members, after informed consent was obtained from all of them. Genomic DNA was isolated from peripheral blood leukocytes using the salting out method. All exons (1–38) and exon–intron boundaries of *CHD7* (ref. seq.: NG_007009.1 and NM_0177880.3) were amplified by polymerase chain reaction using sets of oligonucleotide primers specific for *CHD7* and a thermal cycler (Applied Biosystems, Foster City, CA, USA). Primer sequences and PCR conditions are available on request. PCR products were purified and directly sequenced in both forward and reverse directions on an ABI Prism 3130XL genetic analyzer (Applied Biosystems, Foster City, CA) using the BigDye Terminator Cycle Sequencing Ready Reaction Kit (Applied Biosystems). The very recent discovery of a novel gene associated with ALS, prompted us to search for the presence of mutations in the ALS-linked coding region of *KIF5a*. PCR assay was performed as previously described [26]. The G_4_C_2_-repeat of *C9orf72* was genotyped using a 2-step strategy. First, the repeat number of wild-type alleles was obtained and then it was used a repeat-primed PCR to determine the presence of a G_4_C_2_-expansion as previously described [22, 27]. The G_4_C_2_-repeat expansion, showing the typical saw-tooth pattern, was defined as more than 30 repeats.

### Next generation sequencing

Both patients were screened for mutations in ALS-related genes by means of a targeted next generation sequencing (NGS) analysis. Samples were sequenced with “sequencing by synthesis” approach using the Ion Torrent™ Personal Genome Machine™ (PGM) sequencer (ThermoFisher Scientific) and an Ampliseq™ method with a custom NGS library panel, covering the most known genes implicated in ALS, starting from 50 ng of samples DNA. The custom gene-panel was designed online using the Ion AmpliSeq™ Designer (https://ampliseq.com/browse.action), and resulted in 2-primer pools that are able to amplify 794 amplicons covering all the genes present in the panel, with an amplicon range size of 125–375 bp. In particular, we targeted the coding regions of 39 ALS-related genes including at least 25 bp of intronic flanking regions, and for some selected genes we included the 3′UTR (Supplementary Table 1). The result was a generation of a gene-panel for a total size of 221.88 kb. For the primers pool amplification and the library preparation we used the Ion AmpliSeq™ Library Kit 2.0 following standard protocols, then the libraries was quantified using the Invitrogen™ Qubit™ Fluorometer to determine the dilution factor resulting in a concentration of ~ 100 pM. For the libraries enrichment and the template preparation, an emulsion PCR was used with the Ion PGM™ Hi-Q™ View OT2 Kit on the ION OT2 instrument (ThermoFisher Scientific). The enriched libraries were purified using the Ion OneTouch™ ES, then loaded on a 316-chip with additional other nine samples and sequenced with the Ion PGM™ Hi-Q™ View Sequencing Kit using the ION PGM machine.

### Bioinformatic analysis

Primary bioinformatic analysis (alignment against the GRCh37/hg19 human reference genome, quality and coverage analysis, and variant calling) was performed using the Torrent Suite™ Software (ThermoFisher Scientific), while the VCF files were annotated using wANNOVAR tool (http://wannovar.wglab.org/) and compared against the ExAC database and 1000 Genomes to check the variants frequencies. wANNOVAR program included the use of five functional in silico prediction software programs for non-synonymous variants (PolyPhen-2, SIFT, LRT, MutationTaster, MutationAssessor, FATHMM, CADD, GERP++). To study the number of variants identified in the two samples, the following filter criteria were used: (a) exonic, intronic, 5′UTR, 3′UTR, upstream, downstream, ncRNA exonic and ncRNA intronic; (b) non-synonymous changes; (c) minor allele frequency (MAF) < 0.01 of the European-derived population; (d) variants segregating with the disease phenotype in the family; (e) genotype quality > 75, coverage > 20 obtaining a final variant list with all the variations associated with a rs number, and those variants without any frequencies in the population. The functional annotation of the variants was determined by the prediction software to obtain a prediction of pathogenicity. We considered variants potentially pathogenic if they had a MAF < 0.01 or were predicted to change the amino acid sequence or the splicing junction. Mutations were defined as pathogenic if they had been previously reported in the literature as a causative variant, or if the pathogenicity was confirmed by segregation analysis.

## Results

**Fig. 2:**
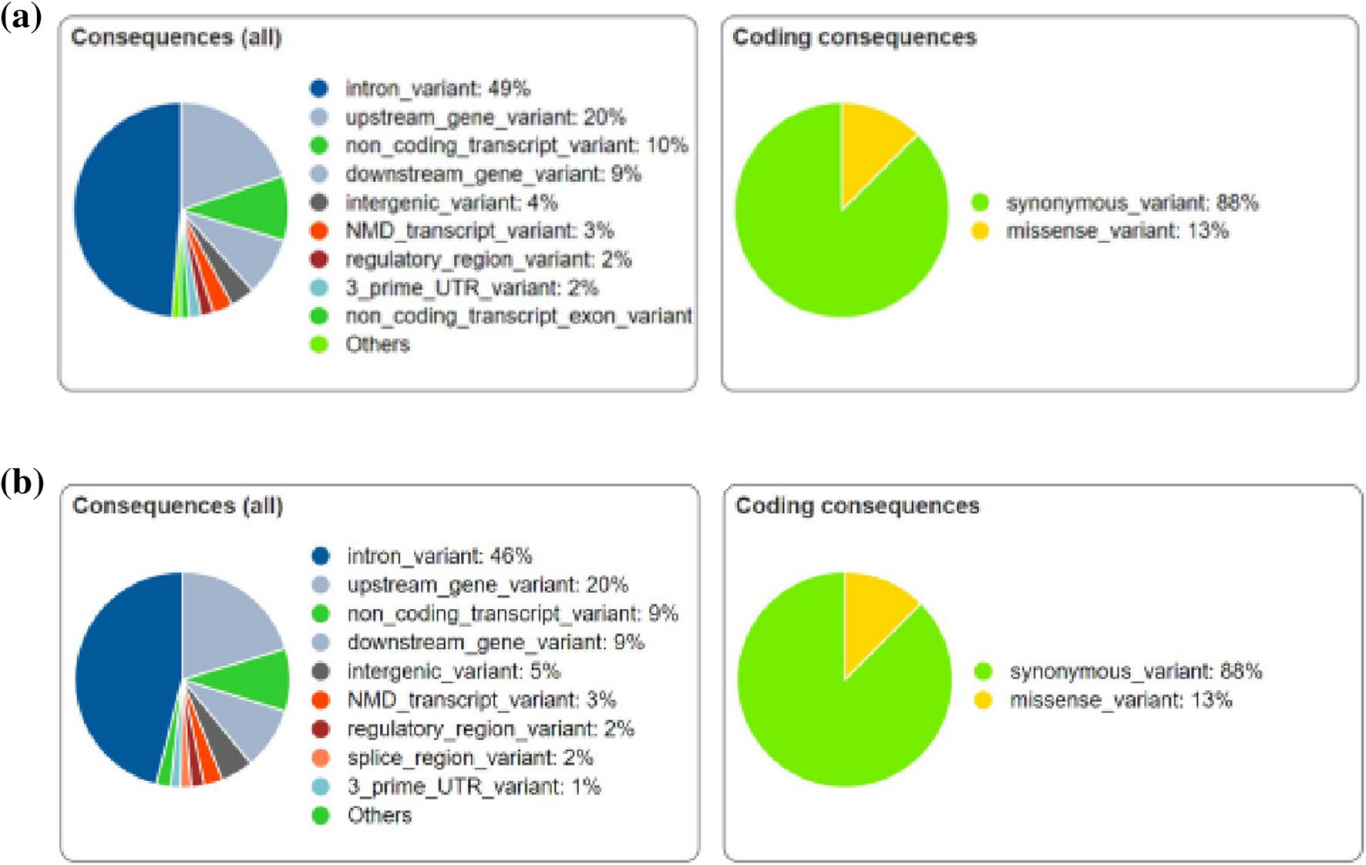
Variant analysis and prediction of the functional consequences of known and unknown variants in ALS (**a**) and CHARGE (**b**) patient by variant effect predictor (VEP) toot (http://www.ensembl.org/)

**Fig. 3:**
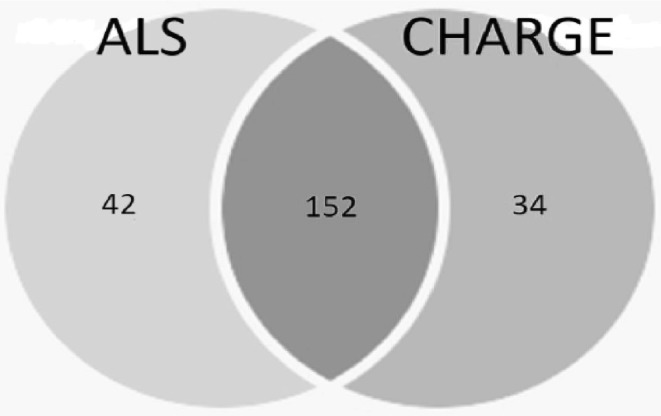
Venn diagram of all variants detected in ALS and CHARGE patients

In the CHARGE patient, molecular analysis of the *CHD7* gene showed a previously described variant in exon 37: c.8016G>A (W2672*, p.Trp2672X). The segregation analysis in the family revealed this variation was absent in the parents, confirming that it was a de novo mutation. This single base exchange was neither found in Exome Aggregation Consortium (ExAC, http://exac.broadinstitute.org/) nor in 1000 genomes database (http://www.1000genomes.org/) and in the Genome Aggregation Database—gnomAD (http://gnomad.broadinstitute.org/); this is a nonsense mutation, leading to the substitution of the Tryptophan 2672 with a premature stop codon. The ALS patient had been screened negative for mutations in *SOD1, TARDBP, FUS*/*TLS* and *C9orf72* genes. The analysis of the ALS-linked coding region of *KIF5A* was negative for any mutations in both sisters (data not shown). Anyway, Targeted Next Generation Sequencing analysis identified known and unknown genetic variations in 39 ALS-related genes. Using a 316 ION Chip, we were able to generate 3,204,815 reads (with a mean reads length of 256 bp) that were aligned to the 99.9% of the Human genome reference 19. The obtained sequencing statistics was: 500,000 mean number reads/sample, 261 (with a range between 81–1150x) mean depth of the 39 analyzed genes with an uniformity between the 93 and 98%. The sequence metrics of both samples were reported in Supplementary Table 2. A total of 380 variants were found (Supplementary Table A; Fig. 2) of which 152 were in common, 42 were detected only in ALS patient, and 34 were reported only in CHARGE patient (Fig. 3). According to the applied filtering strategy and taking into account the *CHD7* mutation found in the CHARGE patient, we focused on unshared variations reported in Supplementary Table B.

## Discussion and conclusions

Here, we gave a clinical and genetic description of two sisters affected by two different disorders: ALS disease and CHARGE syndrome. In our family, one sister, a 43-year-old woman, was clinically ALS diagnosed, with bulbar onset, pyramidal impairment and spastic phenotype. The second one, a full-termed 41-year-old female, carrying the *CHD7* de novo W2672* mutation, presented typical CHARGE syndrome defects. Although a 2–3% recurrence risk is suggested for children of clinically unaffected parents, attributed to parental germline mosaicism [28], we excluded any possible mosaicism in both parents, as revealed by sequence analysis. The mutation, located in the BRK domain of unknown function, was already reported by Jongmans et al. [29], but without any related clinical description and segregation analysis. Anyway, according to data reported in literature by which *CHD7* mutations occur de novo in the vast majority of the typical patients, we assess that the patient carrying this mutation completely fulfill the clinical CHARGE diagnostic criteria [30, 31]. In CHARGE syndrome, basic research has demonstrated that CHD protein complexes affect chromatin structure and gene expression, thus playing an important role in regulating embryonic development; moreover, being assumed CHD7 protein most likely controls gene expression by chromatin remodelling, functioning as a transcription regulator that binds preferentially to methylated histones in enhancer regions and near transcription sites [32], it is clear that CHD7 expression is lowered in the presence of an incomplete and/or not functional protein. Haploinsufficiency for CHD7 is the most likely pathogenic mechanism of this syndrome [33, 34]. Furthermore, epigenetic modifications, including DNA methylation, appear to be involved in motor process influenced by the interaction between genes and environment, and a fraction of those changes might even be transmitted to the offspring [35]. To date, CHARGE and ALS pathologies never occurred together in the same family and no putative correlation between them has been reported on PubMed Central, but whereas many clinical CHARGE features are shared by other syndrome [36–38] we initially hypothesized a possible link between these two diseases. The detection of the *CHD7* de novo mutation in one sister fully explaining her phenotype, disagreed with our starting hypothesis. This evidence prompted us to focus on variants in ALS patient, comparing NGS results between the two sisters. Nonetheless 152 out of the 380 variants were shared by both sisters, this is slightly less than one would expect: we all share 50% of our variants with each of our siblings. That the CHARGE sister has less, might be explained by the fact that she does not have ALS. So the most interesting variants were those that are unique for the ALS patient. Due to roughly 50% of ALS families remain unexplained after routine genetic testing, in addition to ALS caused by mutations in above-mentioned genes, NGS analysis could contribute in identifying rare variants and/or non-coding-variants causing or increasing the risk of the disease. In addition, the very high fold coverage of sequenced fragments obtained by this technology allows for excluding low-grade mosaicism [39]. Anyway, being assumed that genetic aetiology of ALS is responsible for one-third of familial disease, it is unknown how much of the remaining of sporadic cases is genetic and how much is due to other factors such as environmental exposures, aging or lifestyle choices. In CHARGE syndrome, too, epigenetic events have recently emerged as important contributors to the disorder [40]. Anyway, in our case, both in ALS and CHARGE patient NGS analysis revealed no suggestive uncommon variations and no deleterious variants were detected (Supplementary Table B). Indeed, bioinformatical tools (PolyPhen-2, SIFT, LRT, MutationTaster, MutationAssessor, FATHMM, CADD, GERP++) used for analyzing coding missense variants identified in the ALS patient in known ALS genes, predicted no significant scores for damaging effects (data not showed). Among these, we focused on the rs 80,019,660 related to Paraoxonase 1 gene (PON1; c.C602T in exon 6; ref. sEq. NM_000446), the only exonic variant with a MAF of 0.0008. PON 1 has a major protective role both against environmental toxins and as part of the antioxidant defense system and genetic variation across the paroxanase loci may be susceptibility factors for sALS (http://alsod.iop.kcl.ac.uk). Segregation analysis revealed the presence of this polymorphism only in the father of affected sisters. Moreover, the complexity of the genetic architecture of ALS, including an important role for rare genetic variants, has transformed the way we think about this disease. A significant part of ALS heritability cannot be easily explained by only considering a monogenic model, while interactions between multiple ALS genes might explain the considerable phenotypic variability observed among ALS individuals and this leads us to reconsider the traditional classification system for this disease towards a molecular taxonomy for ALS patients’ stratification [41]. To verify whether any genetic variants have any role in the pathogenesis of our ALS patient, further deep investigation by whole genome analysis might be useful.

## Electronic supplementary material

Below is the link to the electronic supplementary material.

Supplementary material 1 (PDF 220 KB)

Supplementary material 2 (PDF 12 KB)

Supplementary material 3 (PDF 71 KB)

Supplementary material 4 (PDF 26 KB)
