# Supplementary material for: ALS and CHARGE syndrome: a clinical and genetic study
Source: Acta Neurol Belg. 2018 Oct 13;118(4):629–35. doi: 10.1007/s13760-018-1029-2 (PMC6244742; doi:10.1007/s13760-018-1029-2)
Supplement: Supplementary file 1 — Supplementary material 1 (PDF 220 KB) [file 13760_2018_1029_MOESM1_ESM.pdf]

Table 1
The 39 ALS-related genes examined in this study

Gene	Location	Name	Gene MIM number	NCBI Reference Sequence	Phenotype	Phenotype MIM number
SOD1, ALS1	21q22.11	Superoxide dismutase 1	147450	NM_000454.4	Amyotrophic lateral sclerosis 1	105400
TARDBP, TDP43, ALS10	1p36.22	TAR DNA-binding protein	605078	NM_007375.3	Amyotrophic lateral sclerosis 10, with or without frontotemporal dementia	612069
					Frontotemporal lobar degeneration, TARDBP-related	612069
FUS, TLS, ALS6, ETM4	16p11.2	Fusion, derived from 12-16 translocation, malignant liposarcom	137070	NM_004960.3	Essential tremor, hereditary, 4	614782
					Amyotrophic lateral sclerosis 6, with or without frontotemporal dementia	608030
ANG, RNASE5, ALS9	14q11.2	Angiogenin	105850	NM_001145.4	Amyotrophic lateral sclerosis 9	611895
CHCHD10, FTDALS2, SMAJ, IMMD	22q11.23	Coiled-coil-helix-coiled-coil-helix domain-containing protein 10	615903	NM_001301339.1	?Myopathy, isolated mitochondrial, autosomal dominant	616209
					Frontotemporal dementia and/or amyotrophic lateral sclerosis 2	615911
					Spinal muscular atrophy, Jokela type	615048
OPTN, GLC1E, FIP2, HYPL, NRP, ALS12	10p13	Optineurin	602432	NM_001008211.1	{Glaucoma, normal tension, susceptibility to}	606657
					Glaucoma 1, open angle, E	137760
					Amyotrophic lateral sclerosis 12	613435

ALS2,<br>*ALSJ,*<br>*PLSJ,*<br>*IAHSP*	2q33.1	Alsin	606352	NM_020919.3	Amyotrophic lateral sclerosis 2, juvenile	205100
					Primary lateral sclerosis, juvenile	606353
					Spastic paralysis, infantile onset ascending	607225
TBK1,<br>*NAK,*<br>*FTDALS4,*<br>*IIAE8*	12q14.2	TANK-binding kinase 1	604834	NM_013254.3	{Encephalopathy, acute, infection-induced (herpes-specific), susceptibility to, 8}	617900
					Frontotemporal dementia and/or amyotrophic lateral sclerosis 4	616439
SPG11,<br>*KIAA1840,*<br>*FLJ21439,*<br>*ALS5,*<br>*CMT2X*	15q21.1	Spastascin	610844	NM_025137.3	Amyotrophic lateral sclerosis 5, juvenile	602099
					Charcot-Marie-Tooth disease, axonal, type 2X	616668
					Spastic paraplegia 11, autosomal recessive	604360
PFN1,<br>*ALS18*	17p13.2	Profilin-1	176610	NM_005022.3	Amyotrophic lateral sclerosis 18	614808
TUBA4A,<br>*TUBA1,*<br>*ALS22*	2q35	Tubulin, alpha-4A	191110	NM_006000.2	Amyotrophic lateral sclerosis 22 with or without frontotemporal dementia	616208
DAO,<br>*DAMOX*	12q24.11	D-amino-acid oxidase	124050	NM_001917.4	{Schizophrenia}	181500
GRN,<br>*CLN11*	17q21.31	Granulin	138945	NM_002087.3	Aphasia, primary progressive	607485
					Ceroid lipofuscinosis, neuronal, 11	614706
					Frontotemporal lobar degeneration with ubiquitin-positive inclusions	607485

SETX, SCAR1, AOA2, ALS4	9q34.13	Senataxin	608465	NM_015046.6	Amyotrophic lateral sclerosis 4, juvenile	602433
					Spinocerebellar ataxia, autosomal recessive 1	606002
TAF15, TAF2N, RBP56	17q12	TAF15 RNA polymerase II, TATA box-binding protein-associated factor, 68kD	601574	NM_139215.2	Chondrosarcoma, extraskeletal myxoid	612237
VCP, IBMPFD1, ALS14, CMT2Y	9p13.3	Valosin-containing protein	601023	NM_007126.4	Inclusion body myopathy with early-onset Paget disease and frontotemporal dementia 1	167320
					Charcot-Marie-Tooth disease, type 2Y	616687
					Amyotrophic lateral sclerosis 14, with or without frontotemporal dementia	613954
UBQLN2, PLIC2, CHAP1, ALS15	Xp11.21	Ubiquilin 2	300264	NM_013444.3	Amyotrophic lateral sclerosis 15, with or without frontotemporal dementia	300857
SQSTM1, P62, PDB3, FTDALS3, NADGP, DMRV	5q35.3	Sequestosome 1	601530	NM_003900.4	Frontotemporal dementia and/or amyotrophic lateral sclerosis 3	616437
					Myopathy, distal, with rimmed vacuoles	617158
					Neurodegeneration with ataxia, dystonia, and gaze palsy, childhood-onset	617145
					Paget disease of bone 3	167250

FIG4, KIAA0274, SAC3, ALS11, YVS, BTOP	6q21	Fig4, S. cerevisiae, homolog of	609390	NM_014845.5	?Polymicrogyria, bilateral temporooccipital	612691
					Amyotrophic lateral sclerosis 11	612577
					Charcot-Marie-Tooth disease, type 4J	611228
					Yunis-Varon syndrome	216340
ANO2, TMEM16B, C12orf3	12p13.31	Anoctamin 2	610109	NM_001278596.1		
APEX, APE	14q11.2	APEX nuclease (multifunctional DNA repair enzyme)	107748	NM_001641.3		
CEP112	17q24.1	Centrosomal protein 112		XM_005257119.5		
CHMP2B, DMT1, VPS2B, ALS17	3p11.2	CHMP family, member 2B	609512	NM_014043.3	Amyotrophic lateral sclerosis 17	614696
					Dementia, familial, nonspecific	600795
CNTF, HCNTF	11q12.1	Ciliary neurotrophic factor	118945	NM_000614.3		
DCTN1, HMN7B	2p13.1	Dynactin 1 (p150, glued, Drosophila, homolog of)	601143	NM_004082.4	Neuropathy, distal hereditary motor, type VIIB	607641
					Perry syndrome	168605
					{Amyotrophic lateral sclerosis, susceptibility to}	105400

HFE, HLA-H, HFE1, MVCD7, TFQTL2	6p22.2	Hemochromatosis gene	613609	NM_000410.3	Hemochromatosis	235200
					[Transferrin serum level QTL2]	614193
					{Alzheimer disease, susceptibility to}	104300
					{Microvascular complications of diabetes 7}	612635
					{Porphyria cutanea tarda, susceptibility to}	176100
					{Porphyria variegata, susceptibility to}	176200
MATR3, MPD2, ALS21	5q31.2	Matrin 3	164015	NM_199189.2	Amyotrophic lateral sclerosis 21	606070
NEFH, CMT2CC	22q12.2	Neurofilament, heavy polypeptide	162230	NM_021076.3	?{Amyotrophic lateral sclerosis, susceptibility to}	105400
					Charcot-Marie-Tooth disease, axonal, type 2CC	616924
PON1, PON, ESA, MVCD5	7q21.3	Paraoxonase-1	168820	NM_000446.6	{Coronary artery disease, susceptibility to}	
					{Coronary artery spasm 2, susceptibility to}	
					{Microvascular complications of diabetes 5}	612633
					{Organophosphate poisoning, sensitivity to}	

PON2	7q21.3	Paraoxonase-2	602447	NM_000305.2	{Coronary artery disease, susceptibility to}	
PRPH	12q13.12	Peripherin	170710	NM_006262.3	{Amyotrophic lateral sclerosis, susceptibility to}	105400
SMN1, SMA1, SMA2, SMA3, SMA4	5q13.2	Survival of motor neuron 1, telomeric	600354	NM_001297715.1	Spinal muscular atrophy-1	253300
					Spinal muscular atrophy-2	253550
					Spinal muscular atrophy-3	253400
					Spinal muscular atrophy-4	271150
SMN2	5q13.2	Survival of motor neuron 2, centromeric	601627	NM_022875.2	{Spinal muscular atrophy, type III, modifier of}	253400
VAPB, VAPC, ALS8	20q13.32	Vesicle-associated membrane protein-associated protein B	605704	NM_004738.4	Amyotrophic lateral sclerosis 8	608627
					Spinal muscular atrophy, late-onset, Finkel type	182980
VEGF, MVCD1	6p21.1	Vascular endothelial growth factor	192240	NM_001171623.1	{Microvascular complications of diabetes 1}	603933
CCNF, FBX1	16p13.3	Cyclin F	600227	NM_001761.2		
NEK1, SRTD6, SRPS2A, ALS24	4q33	Never in mitosis gene A-related kinase 1	604588	NM_001199397.1	{Amyotrophic lateral sclerosis, susceptibility to, 24}	617892
					Short-rib thoracic dysplasia 6 with or without polydactyly	263520
HNRNPA1, IBMPFD3, ALS20	12q13.13	Heterogeneous nuclear ribonucleoprotein A1	164017	NM_002136.3	Amyotrophic lateral sclerosis 20	615426

					?Inclusion body myopathy with early-onset Paget disease without frontotemporal dementia 3	615424
ERBB4, *HER4*, *ALS19*	2q34	Avian erythroblastic leukemia viral (v-erb-b2) oncogene homolog 4	600543	NM_005235.2	Amyotrophic lateral sclerosis 19	615515
