# Supplementary material for: ALS and CHARGE syndrome: a clinical and genetic study
Source: Acta Neurol Belg. 2018 Oct 13;118(4):629–35. doi: 10.1007/s13760-018-1029-2 (PMC6244742; doi:10.1007/s13760-018-1029-2)
Supplement: Supplementary file 2 — Supplementary material 2 (PDF 12 KB) [file 13760_2018_1029_MOESM2_ESM.pdf]

Table 2. Sample sequencing metrics

Sample	Mapped reads	On target	Mean depth	Uniformity	Target base coverage at 20x	Target base coverage at 100x	Amplicons with at least 20 reads	Amplicons with at least 100 reads
ALS	727,468	98,30%	837	93,48%	99.04%	96.58%	99.24%	96.98%
CHARGE	426,89	98,21%	210	95,06%	97.53%	83.58%	98.49%	87.53%
