# Supplementary material for: ALS and CHARGE syndrome: a clinical and genetic study
Source: Acta Neurol Belg. 2018 Oct 13;118(4):629–35. doi: 10.1007/s13760-018-1029-2 (PMC6244742; doi:10.1007/s13760-018-1029-2)
Supplement: Supplementary file 3 — Supplementary material 3 (PDF 71 KB) [file 13760_2018_1029_MOESM3_ESM.pdf]

Table A

List of the variants detected in ALS patient

Chr location	rs ID	Variant	AA change	Position	Gene	ExAC frequency in European
chr1:11072579	rs968545			upstream	TARDBP	.
chr1:11080723	rs70977536			intronic	TARDBP	.
chr1:11082055	rs3835416			intronic	TARDBP	.
chr1:11085004	rs5772439	c.2293_2294insTGTTT		UTR3	TARDBP	.
chr2:202569992	rs3219171			intronic	ALS2	.
chr2:202570232	rs3219170			intronic	ALS2	0.9851
chr2:202574825	rs1210940			intronic	ALS2	.
chr2:202575821	rs3219168	C4015T	L1339L	exonic	ALS2	0.9849
chr2:202575907	rs2882231			intronic	ALS2	.
chr2:202580370	rs3219167			intronic	ALS2	0.7474
chr2:202598113	rs2276615	G2466A	V822V	exonic	ALS2	0.3467
chr2:202606639	rs3731703			intronic	ALS2	.
chr2:202608877	rs4673207			intronic	ALS2	.
chr2:202625615	rs3219156	G1102A	V368M	exonic	ALS2	0.9999
chr2:202626479	.	A238C	S80R	exonic	ALS2	.
chr2:212242192	rs11895168	c.6148T>G		UTR3	ERBB4	.
chr2:212242434	rs7565960	c.5906C>T		UTR3	ERBB4	.
chr2:212242641	rs1595065	c.5699C>T		UTR3	ERBB4	.
chr2:212243075	rs546283804	c.5265C>T		UTR3	ERBB4	.
chr2:212243422	rs1972820	c.4918C>T		UTR3	ERBB4	.
chr2:212243576		c.4764_4762delTGG		UTR3	ERBB4	
chr2:212243582		c.4758_4757delTA		UTR3	ERBB4	
chr2:212243703	rs13003941	c.4637C>A		UTR3	ERBB4	.
chr2:212244761	rs34217661	c.3578_3579insC		UTR3	ERBB4	.
chr2:212244952	rs1836724	c.3388C>T		UTR3	ERBB4	.
chr2:212245090	rs6147150	c.3249_3250insATCCTATTTTCA,		UTR3	ERBB4	.
chr2:212245284	rs12475523	c.3056T>C		UTR3	ERBB4	.
chr2:212245489	rs4672612	c.2851T>C		UTR3	ERBB4	.
chr2:212247834	rs3748960	c.506G>A		UTR3	ERBB4	.
chr2:212251864	rs3748962	A3147G	V1049V	exonic	ERBB4	0.3081
chr2:212252809	rs934607			intronic	ERBB4	.
chr2:212285103	rs2289086			intronic	ERBB4	.
chr2:212285340	rs60420993			intronic	ERBB4	0.0808
chr2:212293044	rs4672613			intronic	ERBB4	.
chr2:212295875	rs6710946			intronic	ERBB4	0.6718
chr2:212543924	rs4673628			intronic	ERBB4	0.0128
chr2:212544024	rs10169970			intronic	ERBB4	.
chr2:212544027	rs67647157			intronic	ERBB4	.

chr2:212587321	rs13002712			intronic	ERBB4	.
chr2:212589986	rs35778743			intronic	ERBB4	.
chr2:212615516	rs6757068			intronic	ERBB4	.
chr2:220118531	rs939574			ncRNA_intronic	TUBA4B	0.8092
chr3:87276571	rs36098294	c.-102C>T		UTR5	CHMP2B	.
chr3:87295049	rs11540913	T189C	T63T	exonic	CHMP2B	0.8974
chr4:170314368	rs7672874			downstream	NEK1	.
chr4:170314951	rs13212	c.710A>G		UTR3	NEK1	.
chr4:170315166	rs1129694	c.495T>C		UTR3	NEK1	.
chr4:170315496	rs4692721	c.165G>A		UTR3	NEK1	.
chr4:170323113	rs4478142			intronic	NEK1	.
chr4:170354836	rs7680152			intronic	NEK1	0.8385
chr4:170354873	rs13139925			intronic	NEK1	.
chr4:170359021	rs6553438			intronic	NEK1	.
chr4:170482883	rs56064008	T1146C	I382I	exonic	NEK1	0
chr4:170506703	rs55679731			intronic	NEK1	0
chr4:170533878				upstream	NEK1	
chr4:170533878				upstream	NEK1	
chr4:170533883				upstream	NEK1	
chr5:138609609	rs11242456			ncRNA_exonic	SNHG4	0.1045
chr5:138613937	rs891994			ncRNA_intronic	SNHG4	.
chr5:138642997	rs59150359	c.-108_-107insT		UTR5	MATR3	.
chr5:138658846	rs2288823			intronic	MATR3	.
chr5:138665756	rs7305	c.672G>A		UTR3	MATR3	0.1480
chr5:138666372	rs10515507			UTR3	MATR3	0.0985
chr5:138667154		c.2070A>C		UTR3	MATR3	
chr5:179259842	rs2450484			intronic	SQSTM1	.
chr5:179260153	rs4935	C876T	D292D	exonic	SQSTM1	0.8512
chr5:179260213	rs4797	G936A	R312R	exonic	SQSTM1	0.8214
chr5:179260494	rs155787			intronic	SQSTM1	.
chr6:26087856	rs2858993			intronic	HFE	.
chr6:26091179	rs1799945	C187G	H63D	exonic	HFE	0.0365
chr6:26091336	rs2071303			intronic	HFE	0.6788
chr6:26094367	rs1572982			intronic	HFE	0.7238
chr6:43738350	rs2010963	c.-94C>G		UTR5	VEGFA	.
chr6:43738977	rs25648	C534T	S178S	exonic	VEGFA	0.0835
chr6:43746169	rs3025000			intronic	VEGFA	0.4108
chr6:43748643	rs3025052			intronic	VEGFA	0.9999
chr6:110106277	rs9320315			intronic	FIG4	0.3951
chr6:110107517	rs9885672	T1961C	V654A	exonic	FIG4	0.4072
chr6:110113765	rs201752617			intronic	FIG4	0.2590
chr6:110146303	rs1127771	G2559A	S853S	exonic	FIG4	0.5766
chr7:94945872	rs854559			intronic	PON1	.
chr7:94946084	rs854560	T163A	L55M	exonic	PON1	0.0365

chr7:94953895	rs705379			upstream	PON1	.
chr7:94953913	rs705380			upstream	PON1	.
chr7:94953949	rs705381			upstream	PON1	.
chr7:95034821	rs9641164			intronic	PON2	0.6401
chr9:35056961	rs1053318	c.153G>T		UTR3	VCP	.
chr9:35060302	rs684562			intronic	VCP	0.0906
chr9:35060746	rs562381			intronic	VCP	.
chr9:35060955	rs2258240			intronic	VCP	0.4680
chr9:35061693	rs6150985			intronic	VCP	0.0898
chr9:35062972	rs514492			intronic	VCP	0.4643
chr9:35068201	rs10972300			intronic	VCP	0.0869
chr9:135138258	rs7025	c.1368G>A		UTR3	SETX	.
chr9:135139000	rs11795382	c.626C>T		UTR3	SETX	.
chr9:135139064	rs11787894	c.562C>A		UTR3	SETX	.
chr9:135139901	rs1056899	A7759G	I2587V	exonic	SETX	0.7330
chr9:135153359	rs35537391			intronic	SETX	.
chr9:135153668	rs11243704			intronic	SETX	0.7205
chr9:135153673	rs11243705			intronic	SETX	0.2923
chr9:135164107	rs2274563			intronic	SETX	.
chr9:135172412	rs2296869	T5811C	D1937D	exonic	SETX	0.7005
chr9:135173454	rs397732808			intronic	SETX	0.6956
chr9:135173685	rs2296871	A5563G	T1855A	exonic	SETX	0.7029
chr9:135202829	rs543573	A4156G	I1386V	exonic	SETX	0.2984
chr9:135203231	rs1183768	G3754A	G1252R	exonic	SETX	0.2982
chr9:135203409	rs1185193	T3576G	D1192E	exonic	SETX	0.3062
chr9:135203530	rs3739922	T3455G	F1152C	exonic	SETX	0.2720
chr9:135205006	rs882709	C1979G	A660G	exonic	SETX	0.4524
chr9:135206460	rs9411449	T1077C	Y359Y	exonic	SETX	0.3022
chr9:135211532	rs7036889			intronic	SETX	.
chr9:135211610	rs111938515			intronic	SETX	.
chr9:135218047	rs11790312			intronic	SETX	0.2727
chr9:135221597	rs497650			intronic	SETX	1
chr9:135230100	rs517076			intronic	SETX	.
chr9:135230222	rs3739917			intronic	SETX	.
chr10:13158262	rs2244380			intronic	OPTN	0.7315
chr10:13166076	rs523747	A964G	K322E	exonic	OPTN	1
chr10:13167860	rs676302			intronic	OPTN	.
chr12:5674754	rs2277398	C2712T	S904S	exonic	ANO2	0.4035
chr12:5721962	rs3741837			intronic	ANO2	.
chr12:5721962	rs3741837			intronic	ANO2	0.7981
chr12:5722181	rs3741838			intronic	ANO2	.
chr12:5757029	rs367206			intronic	ANO2	.
chr12:5841841	rs2277401			intronic	ANO2	0.5734
chr12:5860179	rs2277402			intronic	ANO2	0.6326

chr12:5916350	rs2277403			intronic	ANO2	.
chr12:6030301	rs3741901	C439T	P147S	exonic	ANO2	0.0238
chr12:6054395	rs6489680	c.-12T>C		UTR5	ANO2	0.8333
chr12:64845715	rs61933190			upstream	TBK1	.
chr12:64845867	rs61933191	c.-3784G>A		UTR5	TBK1	.
chr12:64891392	rs57408363			intronic	TBK1	0.0313
chr12:64891393	.			intronic	TBK1	.
chr12:64895984	rs73122364			downstream	TBK1	.
chr12:109278747	rs2111902			intronic	DAO	0.5028
chr12:109281127	rs75861794			intronic	DAO	.
chr12:109281310	rs7980427	G279A	S93S	exonic	DAO	0.0014
chr12:109293320	rs3918347			intronic	DAO	.
chr14:20925154	rs1130409	T444G	D148E	exonic	APEX1	0.4123
chr15:44877629				intronic	SPG11	
chr15:44921073				intronic	SPG11	
chr15:44943757	rs3759871	T1388C	F463S	exonic	SPG11	0.4674
chr16:2485896	rs12928789			intronic	CCNF	0.8737
chr16:2488211	rs12926008			intronic	CCNF	.
chr16:2498828	rs28417759			intronic	CCNF	0.8722
chr16:2498849	rs28670436			intronic	CCNF	0.8729
chr16:2499011	rs8060813			intronic	CCNF	0.8729
chr16:2499786	rs374015103			intronic	CCNF	0.8651
chr16:2500071	rs12919892			intronic	CCNF	.
chr16:31191482	rs929867	c.-54A>G		UTR5	FUS	1
chr16:31195279	rs1052352	C288T	Y96Y	exonic	FUS	0.7513
chr16:31203529	rs4889537	c.770G>C		UTR3	FUS	0.1642
chr17:4852463	rs4790714			upstream	PFN1	.
chr17:34161084				intronic	TAF15	
chr17:34171755		T1443A	G481G	exonic	TAF15	
chr17:34171758	.	C1446T	G482G	exonic	TAF15	0
chr17:34171827	rs4251786	C1515T	Y505Y	exonic	TAF15	0.0002
chr17:42426940	rs9897526			intronic	GRN	0.0929
chr17:42430244	rs5848	c.78C>T		UTR3	GRN	.
chr17:63739352	rs12948946			intronic	CEP112	0.0663
chr17:63822357	rs62065084	A61G	I21V	exonic	CEP112	0.1271
chr17:64001925	rs1015098			intronic	CEP112	0.8758
chr17:64023552	rs12449542			intronic	CEP112	.
chr17:64023624	rs17704679	A1525G	K509E	exonic	CEP112	0.2150
chr17:64023642	rs11079628	T1507C	L503L	exonic	CEP112	0.8612
chr17:64023781	rs11079629			intronic	CEP112	.
chr17:64023800	rs11079630			intronic	CEP112	.
chr17:64024248	rs62063399			intronic	CEP112	.
chr17:64024255	rs56239919			intronic	CEP112	.
chr17:64024303	rs4277386			intronic	CEP112	.

chr17:64024340	rs9901053			intronic	CEP112	.
chr17:64024375	rs9899985			intronic	CEP112	.
chr17:64025331	rs11652766	T1287C	N471N	exonic	CEP112	0.1016
chr17:64025435	rs60700240			intronic	CEP112	.
chr17:64025926	rs9908964			intronic	CEP112	.
chr17:64050007	rs11079637			intronic	CEP112	0.8758
chr17:64050062	rs1401048			intronic	CEP112	.
chr17:64050123	rs8068229			intronic	CEP112	.
chr17:64062699	rs5821547			intronic	CEP112	.
chr17:64062830	rs908713			intronic	CEP112	.
chr20:57022713	rs3069390	c.3422_3429delTGTGTGCA		UTR3	VAPB	0.9091
chr20:57023562	rs4549163	c.4271A>G		UTR3	VAPB	0.0076
chr20:57024541	rs6015274	c.5250T>C		UTR3	VAPB	0.6061
chr22:24108288	rs140182			intronic	CHCHD10	0.6164
chr22:24109550	rs131444			intronic	CHCHD10	0.7191
chr22:24109774	rs179468	A48C	P16P	exonic	CHCHD10	1
chr22:29881884	rs4823040			intronic	NEFH	1
chr22:29885016	rs59371099	G1387A	E463K	exonic	NEFH	0.0005
chr22:29885581	rs267607533	1952_1975del	651_659del	exonic	NEFH	0.0001
chr22:29885599	rs373980795	1970_1975del	657_659del	exonic	NEFH	0.0005
chr22:29885618	rs367989424	T1989A	P663P	exonic	NEFH	0
chr22:29885771		A2142G	E714E	exonic	NEFH	
chr22:29885861	rs165923	T2232C	A744A	exonic	NEFH	0.8192
chr22:29886413	rs165625	A2784G	V928V	exonic	NEFH	0.9230

List of the variants detected in CHARGE patient

**Chr location**	**rs ID**	**Variant**	**AA change**	**Position**	**Gene**	**ExAC frequency in European**
chr1:11072579	rs968545			upstream	TARDBP	.
chr1:11080723	rs70977536			intronic	TARDBP	.
chr1:11082055	rs3835416			intronic	TARDBP	.
chr1:11085004	rs5772439	c.2293_2294insTGTTT		UTR3	TARDBP	.
chr2:202569992	rs3219171			intronic	ALS2	.
chr2:202570232	rs3219170			intronic	ALS2	0.9851
chr2:202574825	rs1210940			intronic	ALS2	.
chr2:202575821	rs3219168	C4015T	L1339L	exonic	ALS2	0.9849
chr2:202575907	rs2882231			intronic	ALS2	.
chr2:202580370	rs3219167			intronic	ALS2	0.7474
chr2:202587583	rs78117864			intronic	ALS2	.
chr2:202598113	rs2276615	G2466A	V822V	exonic	ALS2	0.3467
chr2:202606639	rs3731703			intronic	ALS2	.
chr2:202608877	rs4673207			intronic	ALS2	.

chr2:202625615	rs3219156	G1102A	V368M	exonic	ALS2	0.9999
chr2:212242192	rs11895168	c.6148T>G		UTR3	ERBB4	.
chr2:212242434	rs7565960	c.5906C>T		UTR3	ERBB4	.
chr2:212242641	rs1595065	c.5699C>T		UTR3	ERBB4	.
chr2:212243422	rs1972820	c. 4918C>T		UTR3	ERBB4	.
chr2:212243703	rs13003941	c.4637C>A		UTR3	ERBB4	.
chr2:212244761	rs34217661	c.3578_3579insC		UTR3	ERBB4	.
chr2:212244952	rs1836724	c.3388C>T		UTR3	ERBB4	.
chr2:212245090	rs6147150	c.3249_3250insATCCTATTTTCA		UTR3	ERBB4	.
chr2:212245284	rs12475523	c.3056T>C		UTR3	ERBB4	.
chr2:212245489	rs4672612	c.2851T>C		UTR3	ERBB4	.
chr2:212247834	rs3748960	c.506G>A		UTR3	ERBB4	.
chr2:212251864	rs3748962	A3147G	V1049V	exonic	ERBB4	0.3081
chr2:212252809	rs934607			intronic	ERBB4	.
chr2:212285103	rs2289086			intronic	ERBB4	.
chr2:212293044	rs4672613			intronic	ERBB4	.
chr2:212295875	rs6710946			intronic	ERBB4	0.6718
chr2:212543924	rs4673628			intronic	ERBB4	0.0128
chr2:212544024	rs10169970			intronic	ERBB4	.
chr2:212587321	rs13002712			intronic	ERBB4	.
chr2:220114630	rs138911012	c.1349A>G		UTR3	STK16;TUBA4A	.
chr2:220118531	rs939574			ncRNA_intronic	TUBA4B	0.8092
chr3:87295049	rs11540913	T189C	T63T	exonic	CHMP2B	0.8974
chr4:170314368	rs7672874			downstream	NEK1	.
chr4:170314951	rs13212	c.710A>G		UTR3	NEK1	.
chr4:170315166	rs1129694	c.495T>C		UTR3	NEK1	.
chr4:170315496	rs4692721	c.165G>A		UTR3	NEK1	.
chr4:170323113	rs4478142			intronic	NEK1	.
chr4:170354836	rs7680152			intronic	NEK1	0.8385
chr4:170354873	rs13139925			intronic	NEK1	.
chr4:170359021	rs6553438			intronic	NEK1	.
chr4:170428352	rs370525243			intronic	NEK1	.
chr4:170482883	rs56064008	T1146C	I382I	exonic	NEK1	0
chr4:170506703	rs55679731			intronic	NEK1	0
chr5:138609609	rs11242456			ncRNA_exonic	SNHG4	0.1045
chr5:138613937	rs891994			ncRNA_intronic	SNHG4	.
chr5:138642997	rs59150359	c.-108_-107insT		UTR5	MATR3	.
chr5:138652884	rs181594315			intronic	MATR3	.
chr5:138658846	rs2288823			intronic	MATR3	.
chr5:138665756	rs7305	c.672G>A		UTR3	MATR3	0.1480
chr5:138666372	rs10515507	c.1288T>G		UTR3	MATR3	0.0985
chr5:179259842	rs2450484			intronic	SQSTM1	.
chr5:179260153	rs4935	C876T	D292D	exonic	SQSTM1	0.8512
chr5:179260213	rs4797	G936A	R312R	exonic	SQSTM1	0.8214

chr5:179260494	rs155787			intronic	SQSTM1	.
chr6:26087856	rs2858993			ncRNA_exonic	LOC108783645	.
chr6:26091336	rs2071303			intronic	HFE	0.6788
chr6:26093236	rs1800758			intronic	HFE	0.0465
chr6:26093303	rs1800708			intronic	HFE	0.5685
chr6:26094367	rs1572982			intronic	HFE	0.7238
chr6:43746169	rs3025000			intronic	VEGFA	0.4108
chr6:43748643	rs3025052			intronic	VEGFA	0.9999
chr6:110036274	rs56378532			intronic	FIG4	0.0093
chr6:110053721	rs11153215			intronic	FIG4	.
chr6:110059510	rs2273752			intronic	FIG4	0.3777
chr6:110106234	rs10499054			intronic	FIG4	0.3269
chr6:110110943	rs9384723			intronic	FIG4	.
chr6:110146303	rs1127771	G2559A	S853S	exonic	FIG4	0.5766
chr7:94937419	rs80019660	C602T	A201V	exonic	PON1	0
chr7:94945872	rs854559			intronic	PON1	.
chr7:94946084	rs854560	T163A	L55M	exonic	PON1	0.0365
chr7:94953895	rs705379			upstream	PON1	.
chr7:94953913	rs705380			upstream	PON1	.
chr7:94953949	rs705381			upstream	PON1	.
chr9:35056961	rs1053318	c.153G>T		UTR3	VCP	.
chr9:35060302	rs684562			intronic	VCP	0.0906
chr9:35060746	rs562381			intronic	VCP	.
chr9:35060955	rs2258240			intronic	VCP	0.4680
chr9:35061693	rs6150985			intronic	VCP	0.0898
chr9:35062972	rs514492			intronic	VCP	0.4643
chr9:35068201	rs10972300			intronic	VCP	0.0869
chr9:135137364	rs997784	c.2262A>G		UTR3	SETX	.
chr9:135138258	rs7025	c.1368G>A		UTR3	SETX	.
chr9:135139000	rs11795382	c.626C>T		UTR3	SETX	.
chr9:135139064	rs11787894	c.562C>A		UTR3	SETX	.
chr9:135139195	.	c.431_430delAG		UTR3	SETX	.
chr9:135139901	rs1056899	A7759G	I2587V	exonic	SETX	0.7330
chr9:135146993	rs7046688			intronic	SETX	.
chr9:135150616	rs2296865			intronic	SETX	0.4375
chr9:135152439	rs17148873			intronic	SETX	0.0442
chr9:135153668	rs11243704			intronic	SETX	0.7205
chr9:135153673	rs11243705			intronic	SETX	0.2923
chr9:135164107	rs2274563			intronic	SETX	.
chr9:135172412	rs2296869	T5811C	D1937D	exonic	SETX	0.7005
chr9:135173454	rs397732808			intronic	SETX	0.6956
chr9:135173685	rs2296871	A5563G	T1855A	exonic	SETX	0.7029
chr9:135203530	rs3739922	T3455G	F1152C	exonic	SETX	0.2720
chr9:135203548	.	G3437A	S1146N	exonic	SETX	.

chr9:135203838	rs3739921	C3147T	H1049H	exonic	SETX	0.2371
chr9:135205006	rs882709	C1979G	A660G	exonic	SETX	0.4524
chr9:135211532	rs7036889			intronic	SETX	.
chr9:135218047	rs11790312			intronic	SETX	0.2727
chr9:135221597	rs497650			intronic	SETX	1
chr9:135230222	rs3739917	c.-5407G>A		UTR5	SETX	.
chr10:13158262	rs2244380			intronic	OPTN	0.7315
chr10:13166076	rs523747	A964G	K322E	exonic	OPTN	1
chr10:13167860	rs676302			intronic	OPTN	.
chr11:58391501	rs1800169			ncRNA_intronic	ZFP91-CNTF	0.1430
chr12:5674754	rs2277398	C2712T	S904S	exonic	ANO2	0.4035
chr12:5721962	rs3741837			intronic	ANO2	0.2019
chr12:5721962	rs3741837			intronic	ANO2	0.7981
chr12:5722181	rs3741838			intronic	ANO2	.
chr12:5757029	rs367206			intronic	ANO2	.
chr12:5841841	rs2277401			intronic	ANO2	0.5734
chr12:5860179	rs2277402			intronic	ANO2	0.6326
chr12:5916350	rs2277403			intronic	ANO2	.
chr12:64845715	rs61933190			upstream	TBK1	.
chr12:64845867	rs61933191	c.-3784G>A		UTR5	TBK1	.
chr12:64891392	rs774616946			intronic	TBK1	0.0313
chr12:64891393	rs774616946			intronic	TBK1	0
chr12:64895984	rs73122364			downstream	TBK1	.
chr14:20925154	rs1130409	T444G	D148E	exonic	APEX1	0.4123
chr15:44877650	.			intronic	SPG11	.
chr15:44921069				intronic	SPG11	
chr15:44921066				intronic	SPG11	
chr15:44943757	rs3759871	T1388C	F463S	exonic	SPG11	0.4674
chr15:44955880	rs762777908			upstream	SPG11	0
chr16:2485896	rs12928789			intronic	CCNF	0.8737
chr16:2487224	rs200540114	T441C	P147P	exonic	CCNF	0
chr16:2488211	rs12926008			intronic	CCNF	.
chr16:2498828	rs28417759			intronic	CCNF	0.8722
chr16:2498849	rs28670436			intronic	CCNF	0.8729
chr16:2499011	rs8060813			intronic	CCNF	0.8729
chr16:2500071	rs12919892			intronic	CCNF	.
chr16:31191482	rs929867	c.-54A>G		UTR5	FUS	1
chr16:31195279	rs1052352	C288T	Y96Y	exonic	FUS	0.7513
chr16:31203529	rs4889537	c.770G>C		UTR3	FUS	0.1642
chr17:4848987		c.208T>G		UTR3	PFN1	
chr17:4852463	rs4790714			upstream	PFN1	.
chr17:34161072				intronic	TAF15	
chr17:34161080	rs555404761			intronic	TAF15	.
chr17:34161084				intronic	TAF15	

chr17:34171755		T1443A	G481G	exonic	TAF15	
chr17:34171827	rs4251786	C1515T	Y505Y	exonic	TAF15	0.0002
chr17:42426940	rs9897526			intronic	GRN	0.0929
chr17:63739352	rs12948946			intronic	CEP112	0.0663
chr17:63822357	rs62065084	A61G	I21V	exonic	CEP112	0.1271
chr17:64001925	rs1015098			intronic	CEP112	0.8758
chr17:64023552	rs12449542			intronic	CEP112	.
chr17:64023624	rs17704679	A1525G	K509E	exonic	CEP112	0.2150
chr17:64023642	rs11079628	T1507C	L503L	exonic	CEP112	0.8612
chr17:64023781	rs11079629			intronic	CEP112	.
chr17:64023800	rs11079630			intronic	CEP112	.
chr17:64024248	rs62063399			intronic	CEP112	.
chr17:64024255	rs56239919			intronic	CEP112	.
chr17:64024303	rs4277386			intronic	CEP112	.
chr17:64024340	rs9901053			intronic	CEP112	.
chr17:64024375	rs9899985			intronic	CEP112	.
chr17:64025331	rs11652766	T1287C	N429N	exonic	CEP112	0.1016
chr17:64025435	rs60700240			intronic	CEP112	.
chr17:64025926	rs9908964			intronic	CEP112	.
chr17:64050007	rs11079637			intronic	CEP112	0.8758
chr17:64050062	rs1401048			intronic	CEP112	.
chr17:64050123	rs8068229			intronic	CEP112	.
chr17:64062699	rs753396562			intronic	CEP112	.
chr17:64062830	rs908713			intronic	CEP112	.
chr20:57009796	rs2234487			intronic	VAPB	0.5759
chr20:57013873	rs2234489			intronic	VAPB	.
chr20:57020573	rs1802459	c.1282A>G		UTR3	VAPB	0.2727
chr20:57020760		c.1469_1485delGTGTGTGTGTGTGTGTG		UTR3	VAPB	
chr20:57022713	rs3069390	c.3422_3429delTGTGTGCA		UTR3	VAPB	0.9091
chr20:57024541	rs6015274	c.5250T>C		UTR3	VAPB	0.6061
chr20:57024589	rs6015275	c.5298C>T		UTR3	VAPB	0.2727
chr22:24108288	rs140182			intronic	CHCHD10	0.6164
chr22:24109550	rs131444			intronic	CHCHD10	0.7191
chr22:24109774	rs179468	A48C	P16P	exonic	CHCHD10	1
chr22:29881884	rs4823040			intronic	NEFH	1
chr22:29885016	rs59371099	G1387A	E463K	exonic	NEFH	0.0005
chr22:29885536	.	A1907G	E636G	exonic	NEFH	.
chr22:29885599	rs373980795	1970_1975del	E658_E659del	exonic	NEFH	0.0005
chr22:29885861	rs165923	T2232C	A744A	exonic	NEFH	0.8192
chr22:29886413	rs165625	A2784G	V928V	exonic	NEFH	0.9230
