# Supplementary material for: ALS and CHARGE syndrome: a clinical and genetic study
Source: Acta Neurol Belg. 2018 Oct 13;118(4):629–35. doi: 10.1007/s13760-018-1029-2 (PMC6244742; doi:10.1007/s13760-018-1029-2)
Supplement: Supplementary file 4 — Supplementary material 4 (PDF 26 KB) [file 13760_2018_1029_MOESM4_ESM.pdf]

Table B

List of the variants detected only in ALS patient

Chr location	rs ID	Variant	AA change	Position	Gene	ExAC frequency in European
chr2:202626479	.	A238C	S80R	exonic	ALS2	.
chr2:212243075	rs546283804	c.5265C>T		UTR3	ERBB4	.
chr2:212243576		c.4764_4762delTGG		UTR3	ERBB4	
chr2:212243582		c.4758_4757delTA		UTR3	ERBB4	
chr2:212285340	rs60420993			intronic	ERBB4	0.0808
chr2:212544027	rs67647157			intronic	ERBB4	.
chr2:212589986	rs35778743			intronic	ERBB4	.
chr2:212615516	rs6757068			intronic	ERBB4	.
chr3:87276571	rs36098294	c.-102C>T		UTR5	CHMP2B	.
chr4:170533878				upstream	NEK1	
chr4:170533878				upstream	NEK1	
chr4:170533883				upstream	NEK1	
chr5:138667154		c.2070A>C		UTR3	MATR3	
chr6:26091179	rs1799945	C187G	H63D	exonic	HFE	0.0365
chr6:43738350	rs2010963	c.-94C>G		UTR5	VEGFA	.
chr6:43738977	rs25648	C534T	S178S	exonic	VEGFA	0.0835
chr6:110106277	rs9320315			intronic	FIG4	0.3951
chr6:110107517	rs9885672	T1961C	V654A	exonic	FIG4	0.4072
chr6:110113765	rs201752617			intronic	FIG4	0.2590
chr7:95034821	rs9641164			intronic	PON2	0.6401
chr9:135153359	rs35537391			intronic	SETX	.
chr9:135202829	rs543573	A4156G	I1386V	exonic	SETX	0.2984
chr9:135203231	rs1183768	G3754A	G1252R	exonic	SETX	0.2982
chr9:135203409	rs1185193	T3576G	D1192E	exonic	SETX	0.3062
chr9:135206460	rs9411449	T1077C	Y359Y	exonic	SETX	0.3022
chr9:135211610	rs111938515			intronic	SETX	.
chr9:135230100	rs517076			intronic	SETX	.
chr12:6030301	rs3741901	C439T	P147S	exonic	ANO2	0.0238
chr12:6054395	rs6489680	c.-12T>C		UTR5	ANO2	0.8333
chr12:109278747	rs2111902			intronic	DAO	0.5028
chr12:109281127	rs75861794			intronic	DAO	.
chr12:109281310	rs7980427	G279A	S93S	exonic	DAO	0.0014
chr12:109293320	rs3918347			intronic	DAO	.
chr15:44877629				intronic	SPG11	
chr15:44921073				intronic	SPG11	
chr16:2499786	rs374015103			intronic	CCNF	0.8651
chr17:34171758	.	C1446T	G482G	exonic	TAF15	0
chr17:42430244	rs5848	c.78C>T		UTR3	GRN	.
chr20:57023562	rs4549163	c.4271A>G		UTR3	VAPB	0.0076

chr22:29885581	rs267607533	1952_1975del	651_659del	exonic	NEFH	0.0001
chr22:29885618	rs367989424	T1989A	P663P	exonic	NEFH	0
chr22:29885771		A2142G	E714E	exonic	NEFH	

List of the variants detected only in CHARGE patient

Chr location	rs ID	Variant	AA change	Position	Gene	ExAC frequency in European
chr2:202587583	rs78117864			intronic	ALS2	.
chr2:220114630	rs138911012	c.1349A>G		UTR3	STK16;TUBA4A	.
chr4:170428352	rs370525243			intronic	NEK1	.
chr5:138652884	rs181594315			intronic	MATR3	.
chr6:26093236	rs1800758			intronic	HFE	0.0465
chr6:26093303	rs1800708			intronic	HFE	0.5685
chr6:110036274	rs56378532			intronic	FIG4	0.0093
chr6:110053721	rs11153215			intronic	FIG4	.
chr6:110059510	rs2273752			intronic	FIG4	0.3777
chr6:110106234	rs10499054			intronic	FIG4	0.3269
chr6:110110943	rs9384723			intronic	FIG4	.
chr7:94937419	rs80019660	C602T	A201V	exonic	PON1	0
chr9:135137364	rs997784	c.2262A>G		UTR3	SETX	.
chr9:135139195	.	c.431_430delAG		UTR3	SETX	.
chr9:135146993	rs7046688			intronic	SETX	.
chr9:135150616	rs2296865			intronic	SETX	0.4375
chr9:135152439	rs17148873			intronic	SETX	0.0442
chr9:135203548	.	G3437A	S1146N	exonic	SETX	.
chr9:135203838	rs3739921	C3147T	H1049H	exonic	SETX	0.2371
chr11:58391501	rs1800169			ncRNA_intronic	ZFP91-CNTF	0.1430
chr15:44877650	.			intronic	SPG11	.
chr15:44921069				intronic	SPG11	
chr15:44921066				intronic	SPG11	
chr15:44955880	rs762777908			upstream	SPG11	0
chr16:2487224	rs200540114	T441C	P147P	exonic	CCNF	0
chr17:4848987		c.208T>G		UTR3	PFN1	
chr17:34161072				intronic	TAF15	
chr17:34161080	rs555404761			intronic	TAF15	.
chr20:57009796	rs2234487			intronic	VAPB	0.5759
chr20:57013873	rs2234489			intronic	VAPB	.
chr20:57020573	rs1802459	c.1282A>G		UTR3	VAPB	0.2727
chr20:57020760		c.1469_1485delGTGTGTGTGTGTGTGTG		UTR3	VAPB	
chr20:57024589	rs6015275	c.5298C>T		UTR3	VAPB	0.2727
chr22:29885536	.	A1907G	E636G	exonic	NEFH	.
